# Genetic, maternal, and environmental influences on sociality in a pedigreed primate population

**DOI:** 10.1038/s41437-022-00558-6

**Published:** 2022-09-02

**Authors:** Irene Godoy, Peter Korsten, Susan E. Perry

**Affiliations:** 1 Department of Animal Behaviour, Bielefeld University, Morgenbreede 45, 33615 Bielefeld, Germany; 2 Lomas Barbudal Monkey Project, Proyecto de Monos, Apdo. 5, Bagaces, Guanacaste Costa Rica; 3 Department of Anthropology & Center for Behavior, Evolution, and Culture, University of California—Los Angeles, 341 Haines Hall, 375 Portola Plaza, Los Angeles, CA 90095-1553 USA

**Keywords:** Heritable quantitative trait, Behavioural ecology

## Abstract

Various aspects of sociality in mammals (e.g., dyadic connectedness) are linked with measures of biological fitness (e.g., longevity). How within- and between-individual variation in relevant social traits arises in uncontrolled wild populations is challenging to determine but is crucial for understanding constraints on the evolution of sociality. We use an advanced statistical method, known as the ‘animal model’, which incorporates pedigree information, to look at social, genetic, and environmental influences on sociality in a long-lived wild primate. We leverage a longitudinal database spanning 20 years of observation on individually recognized white-faced capuchin monkeys (*Cebus capucinus imitator*), with a multi-generational pedigree. We analyze two measures of spatial association, using repeat sampling of 376 individuals (mean: 53.5 months per subject, range: 6–185 months per subject). Conditioned on the effects of age, sex, group size, seasonality, and El Niño–Southern Oscillation phases, we show low to moderate long-term repeatability (across years) of the proportion of time spent social (posterior mode [95% Highest Posterior Density interval]: 0.207 [0.169, 0.265]) and of average number of partners (0.144 [0.113, 0.181]) (latent scale). Most of this long-term repeatability could be explained by modest heritability (*h*^*2*^_*social*_: 0.152 [0.094, 0.207]; *h*^*2*^_*partners*_: 0.113 [0.076, 0.149]) with small long-term maternal effects (*m*^*2*^_*social*_: 0.000 [0.000, 0.045]; *m*^*2*^_*partners*_: 0.000 [0.000, 0.041]). Our models capture the majority of variance in our behavioral traits, with much of the variance explained by temporally changing factors, such as group of residence, highlighting potential limits to the evolvability of our trait due to social and environmental constraints.

## Introduction

The last two decades have shown a surge in research linking individual variation in different measures of sociality and variation in survival and/or reproductive success in social mammals (Ellis et al. 2019; Snyder-Mackler et al. 2020), though pathways and mechanisms are poorly understood (Ostner and Schülke 2018; Thompson 2019). In non-human primates, sociality (e.g., dyadic connectedness, social integration) is associated with important measures of biological fitness, including higher offspring survival (Silk et al. 2003, 2009; Cheney et al. 2016; Kalbitzer et al. 2017; McFarland et al. 2017), higher reproductive output (Schülke et al. 2010; Kulik et al. 2012; Langergraber et al. 2013; Feldblum et al. 2021), and increased survival/longevity (Silk et al. 2010; McFarland and Majolo 2013; Archie et al. 2014; Lehmann et al. 2016; Ellis et al. 2019; Campos et al. 2020; Kajokaite et al. 2022). In humans, having stronger social relationships is linked to reduced mortality and improved physical and mental health (Holt-Lunstad et al. 2010). Correlations between sociality and fitness have additionally been shown in a wide range of non-primate mammalian taxa (Yee et al. 2008; Cameron et al. 2009; Barocas et al. 2011; Stanton and Mann 2012; Nuñez et al. 2015; Vander Wal et al. 2015; Ellis et al. 2017; Bond et al. 2021). Identifying how variation in sociality arises, including its genetic basis, is thus relevant to our understanding of the evolution of social behavior.

To understand variation in sociality, we must know what factors influence and constrain it across and within individuals. Importantly, to what extent are measures of sociality repeatable within individuals in the short-term, but also across the lifespan? And what is the relative importance of genetic (e.g., additive genetic) versus non-genetic (e.g., temporal food clustering) sources of variation? Measuring the relative contribution of additive genetic effects is of substantive evolutionary interest, because the heritability of a trait can indicate the potential for a population to respond to selection on it (Falconer and Mackay 1996) (though see (Houle 1992; Morrissey et al. 2010; Hansen et al. 2011)).

Disentangling genetic versus social and other environmental influences on behavior can be difficult in wild animal populations, especially when there is generational overlap and dispersal is limited. For example, determining how parents influence the behavior of offspring in group-living, philopatric species is challenging, because offspring may share social environments (group of residence) in addition to alleles with their parents. Cross-fostering designs can help separate the common environmental versus genetic effects of parents on offspring, but such methods are not feasible with wild primates for both ethical and logistical reasons. Fortunately, in populations where parentage information can be confirmed, or reasonably deduced, alternative approaches are available (Kruuk and Hadfield 2007; Charmantier et al. 2014). The non-genetic and genetic contributions to traits can be assessed through the use of advanced statistical methods known as ‘animal models’ (Kruuk 2004; Wilson et al. 2010; de Villemereuil 2012) that capture genetic variance in a population through the incorporation of relatedness matrices. Narrow-sense heritability (*h*^*2*^) is a statistic that estimates the proportion of total phenotypic variation in a trait in a population that is explained by additive genetic effects (Visscher et al. 2008); in other words, it is the proportion of phenotypic variance due to variance in shared genes (alleles) between individuals in a population. Animal models allow hierarchical structuring of data and inclusion of fixed effects to account for sources of variation (e.g., age, sex) that might otherwise bias heritability estimates. This flexibility likely explains why animal models typically produce more precise estimates for heritability than do alternative methods (e.g., parent-offspring regression) (Postma 2014).

The relative contributions of additive genetic and maternal effects to individual repeatability in behavior have been a topic of much research (Moore et al. 2019)—maternal effects in particular because they can bias estimates of heritability when not adequately controlled for (Rausher 1992), and because they can influence evolutionary responses to selection on traits (Mousseau and Fox 1998; Räsänen and Kruuk 2007). Recent reviews and meta-analyses of animal models show that narrow-sense heritability tends to be low for behavioral traits (at least in comparison to morphological traits) (95% confidence range: 0.092–0.232 (Moore et al. 2019); 95% credible interval: 0.200, 0.271 (Dochtermann et al. 2019)), particularly for social behavior in wild populations (95% credible interval: 0.029, 0.204 (Houslay et al. 2021)). Estimates for maternal effects are even lower (95% confidence range: 0.030–0.098) (Moore et al. 2019). Importantly, most studies on the repeatability of behavior (and their maternal and additive genetic components) are based on short-term observations of individuals only spanning limited parts of their lifespans. For example, less than 10% of the studies reported in a meta-analysis of repeatability of behavior spanned more than one year (Bell et al. 2009). Estimates might not generalize to long-term repeatability across lifespans since repeat measures taken closer in time tend to show higher repeatability than those taken with longer time intervals in between (Bell et al. 2009).

Studies addressing long-term repeatability of social behavior in wild or free-ranging vertebrates across the lifespan are rare (e.g., Aplin et al. 2015; Menz et al. 2017; Houslay et al. 2021; Evans et al. 2021 Strickland et al. 2021), including studies on long-lived primates (Brent et al. 2017; Tkaczynski et al. 2020; Thompson González et al. 2021). In a longitudinal study on free-ranging rhesus macaques, Brent and colleagues (2017) used repeat sampling spanning up to 6 years on individuals to look at repeatability in multiple social network-derived measures of social isolation. Out of five measures, only the amount of grooming given and the amount of grooming received were found to be ‘modestly’ repeatable (i.e., >10% of variance explained by individual identity), with point estimates at 0.24 and 0.183 respectively. In a study on wild chimpanzees, Tkaczynski and colleagues (2020) used repeat sampling spanning up to 19 years for some individuals (mean: 5 years), and found repeatability in aggression, grooming, and spatial association at both the daily (range: 0.05, 0.42) and yearly (range: 0.21, 0.61) level. Maternal effects were not addressed in the study on chimpanzees, whereas in the study on rhesus macaques maternal effects were found to be negligible. Neither study estimated heritability. Here, we present a study estimating long-term repeatability in social behavior in a wild primate population, while also partitioning maternal and additive genetic contributions to repeatability.

We use 20 years of observational data and a multi-generational pedigree from a wild population of white-faced capuchin monkeys (*Cebus capucinus imitator*) to disentangle genetic and non-genetic influences on sociality. White-faced capuchins are a particularly suitable species in which to attempt to partition environmental and genetic influences on a behavioral trait. As a result of long male alpha tenures and high reproductive skew toward alpha males, capuchins have complex social and genetic group structure, with multi-generational overlap and large numbers of close kin through both maternal and paternal lines (Perry 2012; Godoy et al. 2016a). Males tend to co-disperse with close kin (Perry 2012; Wikberg et al. 2014, 2018), and often move to nearby groups. Importantly, these features lead to large numbers of full and half siblings, many of which reside in separate social groups. As a result, capuchin populations consist of individuals that vary in their degree of current and past shared (social) environment as well as relatedness. This leads to a population where high relatedness (*r* > =0.25) is not confined within groups or even matrilines, making it easier to separate out genetic versus non-genetic influences on phenotypic traits.

We focus our analyses on spatial association data, which is perhaps the most widely available type of social data across studies of animal behavior because of common data collection protocols such as the use of instantaneous and scan sampling, where proximity of a subject to other individuals is recorded (Altmann 1974). We expect to find both repeatability and phenotypic plasticity, with social and physical environmental factors playing an important role in the expression of social behavior. In line with findings from recent meta-analyses, we anticipate estimates for heritability and maternal effects to be in the range of low to moderate (<0.3).

## Methods

### Study subjects

Subjects in our study are individually recognized wild capuchins found in and around the Lomas Barbudal Biological Reserve in Guanacaste, Costa Rica. This population has been under observation since 1990 (Perry 2012; Perry et al. 2012), including near continuous observation from January 2002 through March 2020.

### Data collection

We use proximity data on subjects collected during group scan sampling between January 2001 and March 2020 (Altmann 1974). Included in scans are the identity of the subject, and the identity of other individuals within approximately 4 meters of them. Scans have been collected on all individuals in study groups since 2002, and on all adults and subadults since 2001. Scans are taken opportunistically, without regard to time of day. At least 10 min separate consecutive scans of the same individual to reduce the non-independence of scans taken close in time.

Data in this manuscript were collected by 124 observers, with an average of 7.1 data collectors per month. Observers typically work in teams of two to three and rotate across different groups to reduce potential observer bias. Observers also rotate across observer teams to avoid observer drift in coding, since observer teams could potentially start to code behaviors differently from each other in the absence of overlap in observer composition.

### Initial pedigree construction

Of the 376 individuals in our behavioral dataset, 280 (74.5%) were first seen within three months of their births, and we could confidently assign maternity to them based on demographic (pregnancies) and behavioral data (primary nursing) even prior to genotyping. Of the remaining individuals, 41 (10.9%) were males of unknown origin that immigrated into our study population, while the rest were natal to our study groups but were first seen as older infants (>3 months), juveniles, or (sub)adults (14.6%) and required genotyping to assign/confirm maternity. Paternity was assigned based on genetic information when possible (but see *Non-genotyped individuals*).

In total, 287 subjects (76.3%) had two assigned parents, 37 had one assigned parent (9.8%), and 52 (13.8%) had no assigned parent based on demographic, behavioral, and/or genetic parentage information. Most individuals with no assigned parents were immigrant males (78.9%).

#### Genotyping

Information on genetic parentage assignment (at up to 18 microsatellite loci) in our study population is available from previously published work (1996–2005 (Muniz et al. 2006), 2005–2012 (Godoy et al. 2016b)). Partial genotypes (up to 14 loci) have been generated for individuals in this study which more recently entered the study population through birth or immigration (*n* = 91, 2012–2020) (See SI File 1). Briefly, DNA was extracted primarily from non-invasively collected fecal samples, and occasionally from tissue samples obtained from deceased individuals, then amplified at up to 18 autosomal tetranucleotide microsatellite loci (Muniz and Vigilant 2008) using either a 1-step or 2-step PCR protocol (Arandjelovic et al. 2009). There were no significant deviations from Hardy-Weinberg equilibrium, and no evidence of linkage disequilibrium between loci was found (Muniz 2008).

DNA samples were run at a minimum in triplicate, but additional PCRs were performed on low quality samples (e.g., with low quantities of DNA). Genotypes at each of the loci were assigned to be heterozygous when each allele was seen at least twice in independent PCRs, and assigned as homozygous when the allele was seen in at least three independent PCRs in absence of a second allele.

Amplicons were analyzed using an ABI PRISM3100 automated sequencer and GeneMapper Software (Applied Biosystems, Foster City, CA, USA). Likelihood-based parentage assignments were performed using CERVUS 2.0 or 3.0 (Marshall et al. 1998; Kalinowski et al. 2007). The average exclusionary power of the 18 microsatellites was 0.9888 for the first parent and 0.9998 for second parent (Muniz et al. 2006).

Individuals with unknown parents (e.g., immigrant males, founders) were genotyped twice (i.e., using two independent DNA samples) following the procedures described above to guard against sample mix up. Known mother-offspring pairs were confirmed by ascertaining the absence of Mendelian mismatches across all loci for the pair, though one mismatch was allowed to account for null alleles, mutations, and genotyping errors. We detected one null allele in the population in 19 individuals and traced it back to a male who was either the father or grandfather of those individuals (Muniz et al. 2006; Godoy et al. 2016b).

Candidate males for paternity assignment were chosen based on group membership around the time of an infant’s conception (typically 1–10 males). In cases when conceptions occurred prior to the habituation of a study group, we used the identities of all adult males present when the group was first observed. Candidate mothers were similarly chosen for individuals that were first seen as older infants, juveniles, or (sub)adults. For individuals born post-group habituation, CERVUS has always assigned paternity from the pool of potential candidate fathers. Parent-offspring pairs and trios were allowed one mismatch (excluding those at the locus with the known null allele).

### Pedigree updating

#### Non-genotyped individuals

During stable tenures, alpha males in our population sire approximately 73% of infants born in their groups, including 90% of offspring born to unrelated females (Godoy et al. 2016a). There is strong evidence of inbreeding avoidance between alpha males and their female descendants, with relatedness to females as the primary factor constraining alpha male monopolization of paternity within groups (Muniz et al. 2006, 2010; Godoy et al. 2016a, 2016b; Wikberg et al. 2017, 2018). We used this information to update our pedigree, filling missing father information with the identity of the alpha male around the time of a non-genotyped individual’s conception, but only if their mother was not the daughter or granddaughter of the alpha male (i.e., with inbreeding avoidance). This approach allowed us to assign presumed paternity to 21 non-genotyped individuals (5.6% of subjects) who were natal to our study groups.

#### Individuals with missing or incomplete parentage

Out of the original four study groups (from which fissions led to eight additional study groups), we lacked parentage information (i.e., neither parent was sampled) for 12 individuals first seen at the time of habituation. We had incomplete parentage on an additional 11 adults (i.e., only one parent was sampled). We used the software program COLONY version 2.0.6.7 to look for evidence of whether these individuals were related to each other at the level of full sibling (Jones and Wang 2010). We also looked for potential full sibling pairs among the non-natal immigrant males in the population, since co-migrant males are typically kin (Perry 2012; Wikberg et al. 2014, 2018). We assigned five full sibling pairs among co-migrant males, and four full sibling pairs among natal founders. For any remaining co-migrant males and natal founder pairs that were not assigned as full siblings, we assumed these to be either paternal (migrants) or maternal (natal) half siblings, as is typical in this study population (Perry 2012). These assignments are likely to have some error. However, based on what we know about kinship in capuchins, it would introduce more error to assume that these pairs are unrelated.

We pruned our modified population pedigree using the R package *pedantics* version 1.01 (Morrissey and Wilson 2010), to include only individuals that were linked to the subjects in our behavioral dataset. The reduction in missing data can improve convergence and mixing of models (Hadfield 2010). The pruned pedigree contained 419 individuals, with 353 maternities, 354 paternities, 209 full sibships, 413 maternal half sibships, and 1496 paternal half sibships. Maximum pedigree depth was six generations (mean = 3.03).

### Sociality measures (response variables)

**Fig. 1:**
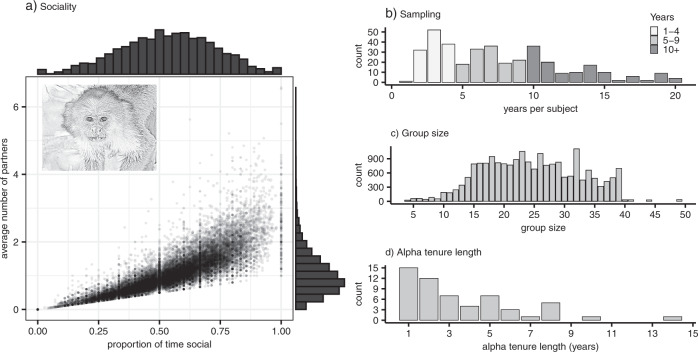
Distribution of sociality, sampling, group size, and alpha tenure length. The scatterplot in **a** shows the proportion of scans per individual per month where the subjects were recorded in proximity of others on the x-axis, and the average number of social partners per scan per month for subjects on the y-axis. The sizes of the circles in **a** are proportional to sample size (range: 5–317 scans per data point). The figure in **b** shows the number of calendar years of data sampling per subject (range: 1–20), **c** variation in group size, and **d** the number of calendar years represented by different alpha tenures in the dataset. Note that **d** does not represent the full diversity of alpha tenure lengths in the population, only within the dataset: some tenure lengths are left-truncated as data from 1990–2000 are not included in this dataset. Figure produced in R using ggplot2 version 3.3.5 (Wickham 2016) and cowplot version 1.1.1 (Wilke 2020). The capuchin image was generated in R using sketcher version 0.1.3 (Tsuda 2020) based on an image taken by Nicholas Schleissmann.

We generated two related proximity-based measures of sociality—(1) whether an individual was seen in proximity of another monkey (within ~4 meters) during a scan (i.e., they were not alone), and (2) the number of partners an individual has nearby (within ~4 meters) during a scan. The former is measure of the propensity of an individual to be social versus alone, while the latter is more indicative of the gregariousness of an individual. These two phenotypes are not independent, as they are generated from the same data (Fig. 1a).

We compiled the scans of individuals by month (mean: 31.9, range: 5–317 scans per month) so that we had counts of (1) the total number of scans where an individual was social and (2) the total number of partners an individual had. With these counts we could look at the (1) proportion of time spent social (versus alone) and (2) the average number of partners an individual had, while still preserving information about sampling density (number of scans).

To be included in any month, subjects needed to have at least five scan samples in that period. As we are interested in the repeatability of our measures of social behavior, subjects had to have at least six months of data to be included.

We excluded dependent infants (less than one year of age) as potential social partners of their mothers. We also excluded these dependent infants as subjects, since an infant is expected to be in close proximity of its mother, particularly during the first half of their first year of life (Godoy 2010; Perry 2012). Including data from infants would likely introduce upward bias to heritability estimates, because mothers and their dependent offspring (whom share high relatedness) would often be in close proximity of each other, and their measures of proximity to others would thus also be highly correlated.

On average, subjects spent just over half of their sampled time within approximately four meters of another monkey (mean: 0.539, standard deviation: 0.193) and had approximately one social partner per scan (mean: 1.057, standard deviation: 0.619) (Fig. 1a). Our dataset consisted of 22,138 monthly sociality scores on 376 subjects generated from 641 140 scans (mean: 56.5 months per subject, range: 6–184 months per subject). Almost all subjects (99.7%, i.e., all but one) were represented by data across more than one calendar year (25, 50, 75% quantiles: 4, 7, 10 different years of data collection) (Fig. 1b).

### Fixed effects

**Fig. 2:**
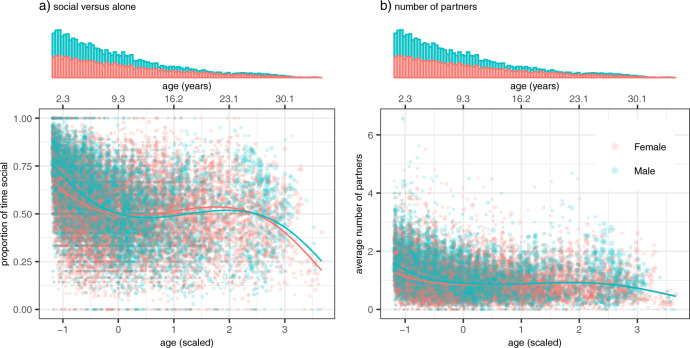
Sociality as a function of age and sex. Circles represent individual monthly data. The sizes of the circles are proportional to sample size (range: 5–317 shows per data point). Circles in **a** represent the proportion of time individuals were seen in proximity of others (not alone) per month, while in **b** represent the average number of partners for individuals per month. Solid lines represent estimated sociality scores based on age and sex, with all other fixed effects set to the mean. The two x-axes represent age as z-scores and in years. Figure produced in R using ggplot2 version 3.3.5 (Wickham 2016).

We included age (as a cubic function) and sex in our models, as well as their interaction to account for differences in how male and female capuchins sexually mature and age. Age in our dataset was right-skewed with higher representation at younger ages (mean: 9.3 years, standard deviation: 6.9) (Fig. 2). To put the ages in developmental context, mean age at first live birth is around 6.3 years for females in this population, though females can begin reproducing in their 5th year (Perry et al. 2012). Males as young as six years old have been known to sire offspring (Godoy et al. 2016b), but males tend to not reach full adult size until their 10th year (Jack et al. 2014).

Seasonal environmental changes, such as in food abundance, or temperature and rain, can lead to changes in how individuals cluster near others, for example, because of how food resources become distributed in the environment. For example, in black-crested gibbons (*Nomascus concolor*), group averages of dyadic proximity have been documented to decrease from the dry season to wet season, with increased average group proximity during cold months and lowered proximity during warm months (Guan et al. 2013). We account for seasonal variation by modelling monthly changes as a sine wave, through inclusion of the sine and cosine functions of a transformed month variable (See SI File 1 for further details).

Central America is a region of ENSO-related precipitation, where the El Niño-Southern Oscillation (ENSO) has an impact on large scale patterns of temperature and precipitation (Ropelewski and Halpert 1987). Bimonthly rainfall anomalies are linked with both the warm El Niño and cool La Niña phases in a neighboring tropical dry forest in Costa Rica, where long-term monitoring of wild white-faced capuchins has shown declines in reproductive output associated with El Niño-like conditions (Campos et al. 2015). To account for the large-scale influence of ENSO on group dynamics, we included a factor variable for three different ENSO phases (Average/Neutral, Cool/La Niña, and Warm/El Niño). We used the bi-monthly Multivariate El Niño/Southern Oscillation (ENSO) index (MEI.v2) obtained from the Physical Sciences Laboratory of the National Oceanic and Atmospheric Administration (https://psl.noaa.gov/enso/mei/, retrieved: 2021-11-06) to determine the different phases. MEI.v2 is a composite index of five different variables (sea level pressure, sea surface temperature, surface zonal winds, surface meridional winds, and Outgoing Longwave Radiation) used to create a time series of ENSO conditions from 1979 to present (Zhang et al. 2019). Warm phases correspond to MEI.v2 values of 0.5 or higher, while cool phases correspond to values of −0.5 or lower.

Demographic differences between groups and within groups across time can also lead to variation in behavior. For example, group size has been found to correlate with the amount of time that individuals spend grooming in various primate species (Dunbar 1991; Lehmann et al. 2007). Group size is also associated with higher sociality measures such as both the number of strong and weak ties that individuals form in diverse clades of primates (Schülke et al. 2022). We attempt to account for variation that arises from such demographic differences by including group size (mean: 24.7, standard deviation: 7.9) (Fig. 1c) as a fixed effect.

In our models, group size and cubic age were centered and scaled to a mean of zero and a standard deviation of one.

### Random effects

All models include the identity of the subject (V_ID_, *n* = 376) as a random factor, as well as subject identity nested within year (V_ID:Year_, *n* = 3150), the identity of each subject’s mother (V_M_, *n* = 142), maternal identity nested within group of residence within year of data collection (V_M:GroupAlpha:Year_, *n* = 2085), and a special variable known as the animal term to account for additive genetic variance (V_A_). These components contribute to long- and/or short-term repeatability of individuals. All models also include year of data collection (V_Year_, *n* = 20), month nested within year (V_Month:Year_, *n* = 224), and the identity of each subject’s group of residence both across years (V_GroupAlpha_, *n* = 56) and within years (V_GroupAlpha:Year_, *n* = 200).

V_ID_ in the models (since the models also additionally estimate V_M_ and V_A_) can be thought of as estimating the “permanent environment variance” (i.e., V_PE_) of an individual, which is the “individual-specific variation in environmental conditions that permanently affect the phenotype (e.g. early-life conditions)” (Dingemanse et al. 2010). V_ID:Year_ captures the variance explained by the repeated sampling of the same individuals within a particular year. We use it to estimate the proportion of the phenotypic variance due to similarity in the trait within individuals from data taken closer in time (within the same year). During such a relatively short period, individuals are more likely to be stable in important social traits such as kin availability, dominance rank for adults, and maternal dominance rank for infants and young juveniles.

V_M_ estimates the variance explained by maternal effects (*m*^*2*^), specifically similarity between maternal siblings. Maternal identities were not available for all subjects, namely 11 immigrant males of unknown origin who were not assigned by COLONY as having a full sibling. We created unique dummy codes for their maternal identities, so that no two of these individuals shared the same mother. We additionally nested maternal identities (V_M:GroupAlpha:Year_) to account for similarity between maternal siblings residing in the same group in the same year. Such a nested structure might capture potential upward biases on heritability due to maternal kin biases in spatial association among siblings residing in the same group.

We estimate *h*^*2*^ in our models by fitting a random effects term (V_A_), referred to as the *animal term*, which in the R package *MCMCglmm* links to the identities of individuals in our population pedigree (Hadfield 2010; see below for details on the implementation of the models in *MCMCglmm*). Inclusion of the animal term provides our models with an additive genetic variance component based on the estimated coefficients of relatedness between individuals in our pedigree. In short, if animals that share more alleles are also more like each other in their behavior, then variation in the behavior may well be due to genetic variation in the population (under the assumption that phenotypic similarity is not due to a shared environment, or is adequately controlled for by fixed and random effects in the model).

V_Year_ and V_Month:Year_ were included in order to account for temporal variation in sociality scores not captured by the fixed effects of seasonality or ENSO phase. These could arise from, for example, observer drift in coding (i.e., measurement error) or prevailing environmental conditions (e.g., drought) that could lead to changes to how individuals cluster near others. There were 218 unique observer combinations across the 224 months represented in the dataset, so V_Month:Year_ should also capture variance due to any differences between observer teams, though we cannot separate out the unique influence of observers.

V_GroupAlpha_ represents variance arising from the different alpha tenures within groups in our study population. V_GroupAlpha_ captures both variance due to group of residence effects and the additional influence of alpha tenures within those groups. In capuchins, alpha males are ‘keystone’ individuals, whose influence is disproportionate relative to that of others in the population, and thus play important roles in establishing group dynamics (Jack and Fedigan 2018). Including group of residence, as defined by alpha tenure, is also important because it helps to account for the higher relatedness within groups within alpha tenures which results from high male reproductive skew toward alpha males. At Lomas Barbudal, males can remain in their alpha position for upwards of 18 years. Alpha tenures in this dataset spanned one to 14 years (Fig. 1d), so we additionally nested the identity of alpha males per group within years (V_GroupAlpha:Year_) so as to separate the within-year and across-year influences of group of residence.

### Statistical methods

We ran analyses in R 4.1.2 (R Core Team 2021), using a Bayesian method with the R package *MCMCglmm* version 2.32 (Hadfield 2010). Data and code used to run all models is provided in the Supplementary Information.

For our binary response variable (social versus alone), which was pooled into monthly units, we fit models with a binomial distribution and logit link function (family = “multinomial2”), with the number of scans each individual was documented social (‘successes’) versus the number of times alone (‘failures’).

For our other response variable (number of partners), which was also pooled into monthly units, we fit models with a Poisson distribution (family = “poisson”), with the total number (sum) of partners per month. We included the natural log of the number of scans per month as a fixed effect to account for sampling effort. We set a strong prior for the log of sampling effort so that the rate at which events occurred was 1 (i.e., we could look at average number of partners per scan).

We used a parameter-expanded prior (V = 1, nu = 1, alpha.mu = 0, alpha.V = 1000) and two inverse Wishart priors (V = 1, nu = 0.002; V = 1, nu = 0.02) for the G structures in our models (i.e., random effects variance components). The prior on the residual variance component was set to one for both the binomial and Poisson models. Estimates for variance components were robust against the choice of prior (SI Fig. 3). We therefore only report findings from models run with parameter-expanded priors in the main text.

Pilot runs (thin = 10, burnin = 3000, nitt = 13,000) indicated that autocorrelation values would remain high for some variance components in models run with parameter-expanded priors, even with large thinning intervals. We therefore increased the number of iterations to guarantee effective sample sizes of at least 1000, but ideally closer to 4000. All models were run with a long burn-in period of at least 10,000 iterations.

We ran multiple chains (*n* = 4) of each model and assessed convergence of the chains visually (SI Files 2a-b), as well as through the Gelman-Rubin criterion implemented via the ‘gelman.diag’ function from the *coda* package in R (version 0.19-4) (Plummer et al. 2006). Scale reduction factors were below 1.02, signifying good convergence. We used Heidelberger and Welch’s convergence diagnostic test for stationarity to check convergence of each chain using the ‘heidel.diag’ function from the *coda* package. Results are presented from the first chain of each model.

#### Reduced models

Inclusion of fixed effects can potentially have an impact on the estimates of variance components in models because total phenotypic variance (V_P_) is estimated (and partitioned among the different random effects) after conditioning on the fixed effects. Heritability estimates, for example, can be higher because the variance explained by the fixed effects structure (V_FE_) is not included in V_P_, thus making the relative contribution of V_A_ to V_P_ larger compared to the same model without fixed effects (Wilson 2008). Conversely, not adequately controlling for relevant fixed effects that contribute to phenotypic variance among and within individuals may potentially lead to an underestimation of V_A_ and associated heritability (*h*^2^).

We ran multiple reduced versions of our models to look at the impact of fixed effects on our variance components. We began with an intercept-only version (i.e., no fixed effects), then built-up complexity by adding in versions with the properties of the individuals first (age, sex), then properties of the group (group size), and subsequently environmental properties (seasonality, ENSO phases). Outputs for these reduced models are provided in the Supplementary Information (SI Table 2, SI Table 3).

We provide the deviance information criterion (DIC) values for models (automatically generated by the *MCMCglmm* package). DIC is a generalization for multi-level models of the Akaike Information Criterion (AIC); and as in AIC, lower DIC values indicate better fit.

#### Transformations from unobserved latent scale to observed data scale

Outputs from our *MCMCglmm* models were on the unobserved latent scale. We used the R package *QGglmm* (version 0.7.4) to additionally compute parameters of interest on the observed data scale (de Villemereuil et al. 2016; de Villemereuil 2018). We used the functions ‘QCicc’ to compute Intra-Class Correlation (ICC) coefficients and ‘QGparams’ to compute additive genetic variance and thus narrow-sense heritability (*h*^*2*^) on the observed data scale. We implemented the ‘QGparams’ and ‘QGicc’ functions with parameters model = ‘binomN.logit’ and n.obs = 32 (the average number of scans per subject per month in our dataset) for the binomial model and model = ‘Poisson.log’ for the Poisson model. The choice of value for n.obs is somewhat arbitrary, and we show the consequences for changes in values of this parameter (i.e., higher estimates with increasing values of n.obs) in SI Fig. 4.

Closed form solutions in *QGglmm* are not available for integrating over posterior distributions generated from binomial models with logit link functions (de Villemereuil 2016). Consequently, using the ‘QGicc’ function is particularly slow. We therefore estimate ICCs from our binomial models using a random subset of the posterior (*n* = 1000 iterations).

The code used for transforming the *MCMCglmm* outputs from the latent scale to the original data scale are available online (see DATA AVAILABILITY).

#### Repeatability and the proportion of variance explained by variance components

Total phenotypic variance (V_P_) was the sum of estimates from all variance components and residual variance in a model (V_P_ = V_ID_ + V_ID:Year_ + V_M_ + V_M:GroupAlpha:Year_ + V_A_ + V_GroupAlpha_ + V_GroupAlpha:Year_ + V_Month:Year_ + V_Year_ + V_residual_). The proportion of variance explained by each variance component was calculated by including its estimate in the numerator while including total phenotypic variance in the denominator. So, for example the proportion of variance explained by year of data collection was calculated as $\left( {\frac{{V_{Year}}}{{V_P}}} \right)$.

Long-term repeatability was calculated with the sum of V_ID_, V_M_, and V_A_ in the numerator. Short-term repeatability was calculated similarly but with inclusion of within-series variances (V_ID_ + V_M_ + V_A_ + V_ID:Year_ + V_M:GroupAlpha:Year_) in the numerator to capture additional consistency in among-individual differences resulting from greater environmental similarity within a time series (i.e., year).

We report posterior modes and 95% Highest Posterior Density intervals (i.e., 95HPDI in square brackets). Unless mentioned otherwise, we present results on the unobserved latent scale, and without the variance from the fixed effects (V_FE_) incorporated into V_P_. For completeness, estimates with V_FE_ included in V_P_ and transformations to the observed data scale are also provided in SI Table 3.

## Results

### Fixed effects

**Fig. 3:**
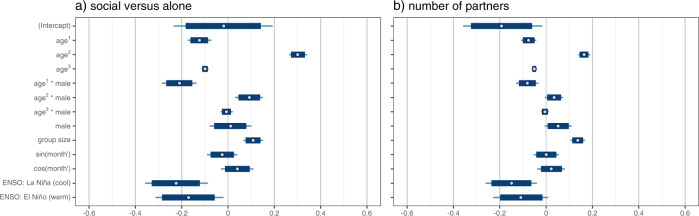
Uncertainty intervals for fixed effects. All models were run in quadruplicate to check for convergence. Posterior draws came from all four chains merged. Point estimates are posterior means. The thin lines show 99% posterior intervals, while the thick boxes show 95% posterior intervals. Figure produced in R using bayesplot version 1.8.1 (Gabry 2020), patchwork version 1.1.1 (Pedersen 2020), and Cairo version 1.6-0 (Urbanek and Horner 2020).

Group size, ENSO phases, and the interaction between age and sex had effects on the likelihood that an individual was in proximity of others, both for time spent social versus alone and the number of partners (Fig. 3, SI Table 2). Sociality decreased with age for both sexes, with males initially slightly more social than their female counterparts (Fig. 2). Sociality increased with group size and decreased both during La Niña and El Niño phases (Fig. 3). Effects of seasonality were weak; point estimates for both the sine and cosine transformation of month were near zero, and the 95% credible intervals for their estimates overlapped with zero (Fig. 3).

### Variance components

The posterior distributions (latent scale) for V_ID_, V_ID:Year_, V_M_, V_M:Group:Year_, V_A_, V_Year_, V_Month:Year_, V_GroupAlpha_, and V_GroupAlpha:Year_ showed peaks distinct from zero for both behavioral measures. However, V_M_ estimates had high density near zero for both behavioral measures, and V_ID_ estimates had high density near zero for number of partners but not time spent social (SI File 2a and SI File 2b).

**Fig. 4:**
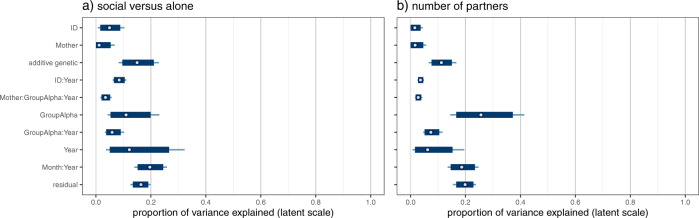
Uncertainty intervals for proportion of variance explained by the random effects. Output is on the unobserved latent scale, and the variance explained by the fixed effects is not included in the total phenotypic variance. All models were run in quadruplicate to check for convergence. Posterior draws came from all four chains merged. Point estimates are posterior means. The thin lines show 99% posterior intervals, while the thick boxes show 95% posterior intervals. Figure produced in R using bayesplot version 1.8.1 (Gabry 2020) and patchwork version 1.1.1 (Pedersen 2020).

Estimates for the proportions of variance explained by the random effects were similar in both models, but usually with higher estimates in the model for time spent social than for number of partners (Fig. 4, SI Table 3).

### Repeatability, heritability, and maternal effects

**Fig. 5:**
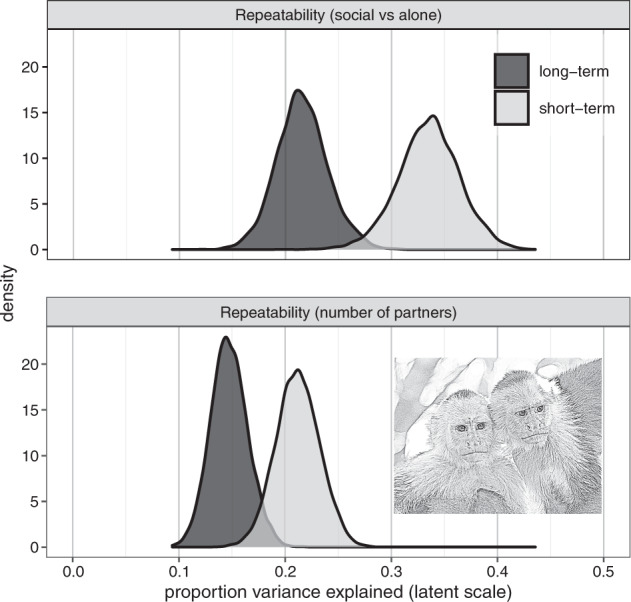
Posterior distributions for repeatability estimates. Output is on the unobserved latent scale, and the variance explained by the fixed effects is not included in the total phenotypic variance. All models were run in quadruplicate to check for convergence. The output presented is from the first chain for each model. Figure produced in R using ggplot2 version 3.3.5 (Wickham 2016). The capuchin image was generated in R using sketcher version 0.1.3 (Tsuda 2020) based on an image taken by Nicholas Schleissmann.

There was modest (>0.1) long-term repeatability $\left( {\frac{{V_{ID} \,+\, V_M \,+\, V_A}}{{V_P}}} \right)$ in both the amount of time spent social (*R*_*long-term*_: 0.207 [0.169, 0.266]) and the number of partners (*R*_*long-term*_: 0.144 [0.113, 0.181]) (Fig. 5). Repeatability was largely attributed to additive genetic effects $\left( {\frac{{V_A}}{{V_P}}} \right)$ (0.152 [0.094, 0.207]; *h*^*2*^_partners_: 0.113 [0.076, 0.149] (Table 1), while estimates were low for maternal effects $\left( {\frac{{V_M}}{{V_P}}} \right)$ (*m*^*2*^_social_: 0.000 [0.000, 0.045]; *m*^*2*^_partners_: 0.000 [0.000, 0.041]) and permanent environment effects $\left( {\frac{{V_{ID}}}{{V_P}}} \right)$ (social: 0.050 [0.015, 0.087]; partners: 0.000 [0.000, 0.033]).

**Table 1:** Heritability estimates.

Behavior	Scale	V_FE_ accounted for with V_P_	95HPDI		
			*h*^*2*^	lower	upper
social versus alone	latent	No	0.152	0.094	0.207
–	–	Yes	0.096	0.068	0.154
–	data	No	0.110	0.068	0.151
–	–	Yes	0.079	0.055	0.122
number of partners	latent	No	0.113	0.076	0.149
–	–	Yes^a^	0.094	0.065	0.125
–	data	No	0.022	0.015	0.030
–	–	Yes^a^	0.024	0.016	0.030

Posterior modes and 95% highest posterior density intervals for heritability estimates.

^a^V_FE_ does not include log(n), which is an offset term for sampling effort.

Short-term repeatability $\left( {\frac{{V_{ID} \,+\, V_{ID:Year} \,+\, V_M \,+\, V_{M:GroupAlpha:Year} \,+\, V_A}}{{V_P}}} \right)$ explained approximately a third of the variation in time spent social (R_social_: 0.343 [0.278, 0.391]), but less for number of partners (R_partners_: 0.213 [0.172, 0.252]) (Fig. 5). Maternal effects remained low when adding within-in year contributions $\left( {\frac{{V_M \,+\, V_{M:GroupAlpha:Year}}}{{V_P}}} \right)$ (social: 0.045 [0.023, 0.085]; partners: 0.044 [0.022, 0.073]).

### Group of residence

Group of residence across years, as defined by alpha male tenures within groups (V_GroupAlpha_), explained modest variation in time spent social (0.108 [0.043, 0.184]) and a larger proportion of variance in number of partners (0.253 [0.164, 0.367]). A smaller additional amount of variation was explained by alpha tenures within groups within years (V_GroupAlpha:Year_) for both time spent social (0.061 [0.036, 0.089]) and number of partners (0.071 [0.051, 0.104]).

Overall, the effect of group of residence (as defined by alpha tenures within groups) $\left( {\frac{{V_{GroupAlpha} \,+\, V_{GroupAlpha:Year}}}{{V_P}}} \right)$ explained about twice as much variance in number of partners (0.332 [0.245, 0.436]) as it did for time spent social (0.163 [0.108, 0.246]).

### Month and year of sampling

V_Month:Year_ explained modest variation in both time spent social (0.208 [0.152, 0.245]) and number of partners (0.179 [0.146, 0.234]). V_Year_ explained modest variation in time spent social (0.110 [0.038, 0.240]), but had lower estimates for number of partners (0.056 [0.009, 0.138]). Estimates for V_Year_ were more uncertain (wide HPD intervals).

### Effect of inclusion of fixed effects on variance components

**Fig. 6:**
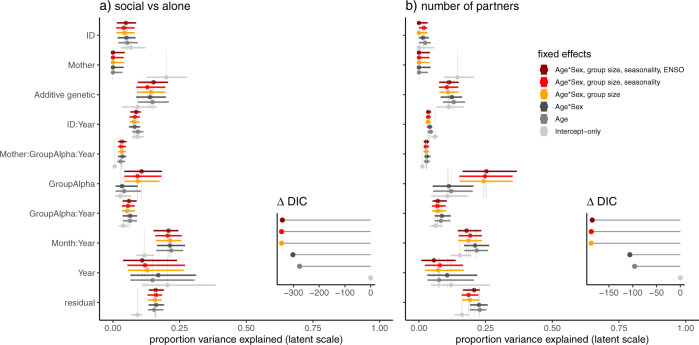
Proportion of variance explained by the random effects, categorized by the fixed effects structure of each model. Output is on the unobserved latent scale, and the variance explained by the fixed effects is not included in the total phenotypic variance. The lollipop dot plots show changes in DIC scores with increasing model complexity (i.e., number of fixed effects in the models). All models with number of partners as the response (the Poisson models) also include the natural log of the numbers of group scans (i.e, an offset for sampling effort). All models were run in quadruplicate to check for convergence. The output presented is from the first chain for each model. Figure produced in R using ggplot2 version 3.3.5 (Wickham 2016) and cowplot version 1.1.1 (Wilke 2020).

The proportion of variance explained by each random effect did not change dramatically with changes to the fixed effects structure of the models, with the exception of V_M_, V_GroupAlpha_, and V_Month:Year_ (Fig. 6). Estimates for V_M_ were large when no fixed effects were included but were low and close to zero once age was controlled for. In contrast, V_M:GroupAlpha:Year_ increased slightly with the inclusion of fixed effects, though estimates always remained low. Estimates for group of residence effects across years were larger once group size was accounted for, while estimates for V_Month:Year_ were lowest in the absence of any fixed effects but similar once age, at least, was accounted for. The largest changes to DIC scores for models came with inclusion of age and group size (Fig. 6).

## Discussion

Disentangling genetic and non-genetic sources of variation in social behaviours is challenging, in part because closely related individuals often have shared social environments. Using quantitative genetic animal models, we combine a multi-generational pedigree and two decades of observational data on wild capuchin monkeys, to partition genetic, social, and environmental sources of variation on spatial proximity-based measures of sociality. We find ‘modest’ (>0.1) repeatability in sociality, which is largely explained by additive genetic sources of variation, and to only a small extent by shared maternal environments between siblings. Much of the variance in our models is explained by temporally changing factors such as group of residence, month, and year of data collection.

Our posterior mode estimates for repeatability (short-term) in time spent social (0.343) and number of partners (0.213) fall in the range reported in a meta-analysis by Bell and colleagues (2009), where the modal repeatability value of behavioral traits was found to be around 0.2 (mean: 0.37). With sampling across multiple years for most subjects (10+ different calendar years for 33.3% of subjects), we were able to separate out long-term (across years) repeatability from this short-term repeatability. Separating long-term from short-term repeatability allowed us to capture and account for autocorrelation or dependency of observations taken closer in time (within the same calendar year) (Araya-Ajoy et al. 2015). The within-year contributions to repeatability were small relative to the across-year contributions. Individual capuchins thus seem consistent in their spatial association patterns across the years.

Repeatability was largely accounted for by additive genetic sources of variation, though non-zero maternal effects indicate there is similarity between siblings beyond that caused by additive genetic effects. However, these maternal effects were small and largely limited to within-year variation. Our point estimates for maternal effects (*m*^*2*^_*social*_: 0.045; *m*^*2*^_*partners*_: 0.044) and heritability (*h*^*2*^_*social*_: 0.152; *h*^*2*^_*partners*_: 0.113) are within the synthetic range (95 CI, *m*^*2*^: 0.030, 0.098; *h*^*2*^: 0.092, 0.232) reported for behavioral traits (Moore et al. 2019). Our results are comparable to those reported by Moore and colleagues, as the studies they analyzed also partitioned maternal and additive genetic sources of variation within the same models. Findings of low maternal effects on offspring behaviour are often surprising to those researching species with prolonged maternal care like capuchins; however, maternal effects generally tend to explain half as much variance in phenotypic traits as do additive genetic effects (Moore et al. 2019).

Estimates of heritability can be upwardly biased when closely related individuals share social environments not accounted for (e.g., estimating V_A_ without estimating V_M_). Similarly, maternal effects can be inflated when not accounting for shared genes between siblings (e.g., estimating V_M_ without estimating V_A_). Likewise, in assessing both heritability and maternal effects it is useful to consider other common environment effects that may also bias estimates. One such potential source is paternal effects. In capuchins, such influences would best be captured by the identity of alpha males during an individual’s infancy. This is because alpha males are typically the adult male around whom infants spend the most time (Godoy et al. 2016b) and have close interactions with (Sargeant et al. 2016), and these males are also likely to be the father or grandfather of the infants (Jack and Fedigan 2006; Muniz et al. 2006, 2010; Godoy et al. 2016a, 2016b; Wikberg et al. 2017, 2018). While we did not account for paternal effects directly, these are likely captured in part by another variance component in our models, V_GroupAlpha_, which represents variance due to the identity of the alpha males nested within the groups. Our data are heavily skewed toward younger individuals, and in these younger individuals, the current alpha male is likely to be the same as at the individuals’ birth. V_GroupAlpha_ should thus capture some, though not all, social father effects, reducing potential inflation of our V_A_ estimates due to shared paternal environments.

Most phenotypic variance in our models could be explained by non-intrinsic differences between individuals (i.e., factors other than genetic, long-term maternal, and permanent environmental effects), reflecting short-term phenotypic plasticity in our behavioral measures, and environmental (social and/or physical) influences. Group of residence (defined by alpha tenures within groups) explained a modest proportion of variance in time spent social (0.163) and even more for number of partners (0.332). Groups thus vary consistently in their sociality, particularly regarding the average number of partners; or in other words, some groups are consistently more social and gregarious across years than other groups. These differences are not driven by differences in group sizes, which are already accounted for in the models (by including group size as a fixed effect). In fact, not including group size reduces estimates for group of residence. These stable group differences may be consequential if, for example, more social groups can outcompete less social groups. Future studies on sociality should therefore consider not just individual-level fitness consequences of sociality, but also group-level consequences which may well amplify or constrain selection for more social individuals.

The variance explained by the factor Month:Year (V_Month:Year_) (mode_social_: 0.208; mode_partners_: 0.179) may reflect the influence of shorter term temporal changes in clustering of food sources (e.g., fruiting fig trees), while V_Year_ (mode_social_: 0.110; mode_partners_: 0.056) may reflect more long-lasting environmental changes (e.g., drought), though both may also reflect observer bias or drift in coding (i.e., measurement error). More direct methods for assessing environmental influences (e.g., measuring food clustering) while controlling for observer identities would be necessary to better understand what drives consistency in behavioral measures within years and months.

Seasonality had little impact on sociality, whereas there was stronger evidence for the influence of ENSO phases, group size, and the interaction between age and sex on both time spent near others and number of partners. Sociality decreased with increasing age. Juvenile males were more social than female juveniles, particularly early in development, but these differences disappeared by adulthood. An adult (age 9.3 years) from a large group (1 SD above mean, ~33 individuals) was on average more social than an individual from a small group (1 SD below mean, ~17 individuals), both when sociality was measured as (a) proportion of time spent social (large: 0.52, small: 0.47) and (b) average number of partners (large: 0.95, small: 0.72) though the differences were not dramatic. El Niño (dry) and La Niña (wet) phases were both associated with reduced sociality scores. Drought conditions during El Niño phases likely drive individuals farther apart as they spend more time searching for scarcer resources. La Niña phases may also lead to changes in how individuals cluster near others. However, since heavy rainfall also affects visibility in the field, the effects captured by La Niña phases may also reflect underestimation of sociality due to reduced visibility in the canopy. Observers are less able to accurately count the number of conspecifics near a subject when visibility is poor.

The inclusion of age and of group size had the largest impact on model fit and estimates for the relative contribution of variance components. However, estimates for variance components were largely robust to changes in the fixed effects structure of models, with little difference between reduced and full models once age, at least, was accounted for. Repeatability was higher in models lacking any fixed effects, driven by much larger maternal effects (which disappeared with inclusion of age as a fixed effect). In other words, the effect of individual age, if not accounted for, is associated with increased similarity between the social phenotypes of maternal siblings. Inclusion of age increased estimates for month of data collection for both phenotypes and also increased heritability estimates for time spent social, though there was little change across models for number of partners. Large maternal effects in the intercept-only models may reflect the age structure of our dataset, which is highly skewed toward younger individuals. Also, in the absence of controlling for the effects of age, individuals may appear more consistent in their behavior simply because they are observed more intensely over a shorter range of time. This may be compounded by higher maternal effects in younger age groups, since maternal effects are typically stronger in juvenile versus adult stages of development (Moore et al. 2019). Other fixed effects had smaller influences on estimates for the variance components. The influence of group of residence (alpha tenure within group) across years increased once group size was accounted for.

A limitation in understanding sources of variation in our behavioral traits is that the phenotype of one individual can be influenced by the genes expressed in others. The social environment in which an individual finds itself is an aggregate of the expression of genes from potential interaction partners. These indirect genetic effects are a source of genetic variation that can both constrain or facilitate an evolutionary response to selection of a trait (Moore et al. 1997; Wolf et al. 1998; Bijma and Wade 2008). In considering the heritability of a behavioral trait in a highly social animal, such as a capuchin, it is important to consider the influence of the genetic social environment (i.e., the variation in genes of the pool of potential interacting partners). We have not addressed indirect genetic effects in the current study but hope to explore their influence in future studies.

Another related limitation in our study, and in most studies on uncontrolled populations, is the non-independence of data points collected on social behavior. This issue is particularly problematic in capuchins, since they show strong maternal kin-biases in affiliation (Perry et al. 2008; Kalbitzer et al. 2017). To partially deal with this issue, we did not include dependent infants in our study, as their spatial association data would largely reflect that of their mothers and would thus be expected to inflate heritability estimates. Unfortunately, we cannot fully account for potential biases introduced from strong affiliative ties between mother-offspring pairs past infancy, which is a general limitation in populations with prolonged mother-offspring bonds. We did, however, look for other potential sources of maternal kin biases in the data. Our models show little similarity among maternal siblings across years (V_M_ estimates close to zero). Those estimates might be low because many maternal sibling pairs do not reside in the same group. We thus additionally nested maternal identities within group within year (V_Mother:GroupAlpha:Year_), to capture potential biases that might arise from maternal siblings spending lots of time near each other (siblings living in the same group in the same year). Nesting maternal effects within group of residence and year of data collection captured only a small proportion of the total variance in our models, giving us more confidence that maternal kin biases in spatial association were not upwardly biasing our estimates for heritability.

Conditioned on the effects of sex, age, group size, seasonality, and ENSO phases, our models captured most of the variance in our behavioral traits (latent scale). Much of that variance was explained by temporally changing factors, which are in addition to additive genetic, long-term maternal, and permanent environmental effects. Our results highlight potential limits to the evolvability of our behavioral trait, because of constraints imposed by social (e.g., group of residence) or other temporally changing factors (e.g., month and year effects). We provide one of the first longitudinal studies to disentangle genetic and non-genetic influences on social behavior in a long-lived primate, while thoroughly attempting to account for common environmental effects. Studies on primates are often more focused on social or environmental effects on sociality, in particular maternal effects given the extended period of maternal care. Our results indicate that genetic effects may be just as (or more) critical than social parental effects in determining long-term repeatability, at least in measures of spatial association. Extensive pedigree and longitudinal social phenotype data are available from several long-term primate studies. These data, in combination, are key for studying the evolution of sociality. Extensive pedigrees have been used to look at, as examples, the contribution of additive genetic effects to the gut microbiome (Grieneisen et al. 2021) and for estimating rates of contemporary adaptive evolution in wild animal populations (Bonnet et al. 2022). We hope that other long-term field studies join in further investigating the relative importance of genetic versus non-genetic effects in driving variation in sociality, as this information is crucial to understanding how a population can respond to selection and thus the potential for adaptive evolution in sociality.

## Supplementary information

Supplementary Information File 1

Supplementary Table 3

## Data Availability

Data files with all outcome and predictor variables, as well as the R code used to run analyses, are available on Dryad 10.5061/dryad.mkkwh7136.
