# Supplementary material for: Genetic, maternal, and environmental influences on sociality in a pedigreed primate population
Source: Heredity (Edinb). 2022 Sep 2;129(4):203–14. doi: 10.1038/s41437-022-00558-6 (PMC9519975; doi:10.1038/s41437-022-00558-6)
Supplement: Supplementary file 1 — Supplementary Information File 1 [file 41437_2022_558_MOESM1_ESM.docx]

**Supplementary Information for**

**Genetic, maternal, and environmental influences on sociality in a pedigreed primate population**

Irene Godoy^1,2^, Peter Korsten^1^, and Susan E. Perry^2,3^

^1^Department of Animal Behaviour

Bielefeld University

Morgenbreede 45
33615 Bielefeld, Germany

^2^Lomas Barbudal Monkey Project

Proyecto de Monos

Apdo. 5

Bagaces, Guanacaste, Costa Rica

^3^Department of Anthropology & Center for Behavior, Evolution, and Culture

University of California - Los Angeles

341 Haines Hall, 375 Portola Plaza

Los Angeles, CA 90095-1553, USA

Correspondence: I. Godoy. (email: [irene.godoy@gmail.com](mailto:irene.godoy@gmail.com))

**Genotyping (samples from 2012-2020)**

Sample collection and field processing

Fecal samples were collected non-invasively from individually recognized capuchins using a two-step method involving ethanol and silica (Nsubuga et al. 2004; Roeder et al. 2004). Up to 5 grams were collected within 10 minutes of the subject defecating and placed immediately into a 50-mL conical tube containing 30 mL of a 97% ethanol solution. The samples were kept in ethanol for at least 24 hours and then stored at 4° or -20° C, depending on space availability. The ethanol solution was then drained, and the remaining fecal matter was transferred to a new 50 mL conical tube containing 20 grams of dry silica beads. Silica-preserved samples were stored at room temperature until shipment to the Max Planck Institute for Evolutionary Anthropology (MPI-EVA) in Leipzig, Germany, where they were stored at 4°C until further processing.

DNA extraction and genotyping

DNA was extracted from up to 100 mg of fecal matter using QIAamp DNA Stool Mini Kits (Qiagen), with slight modification of the manufacturer’s instructions (Morin et al. 2001). DNA was eluted with water to a final volume of 200 uL.

DNA was amplified using 18 tetranucleotide microsatellite markers (Muniz and Vigilant 2008), which were previously used to successfully genotype 334 capuchins from our study population (Muniz et al. 2006; Godoy et al. 2016). We additionally included three sex-specific markers which have previously been used in Platyrrhini (UTX, UTY2, SRY (Di Fiore 2005; Villesen and Fredsted 2006; Cadamuro et al. 2015)), as well as two additional tetranucleotide autosomal markers (d3s1766, d12s372).

Genotyping was accomplished used a two-step multiplex polymerase chain reaction (PCR) method (Arandjelovic et al. 2009), modified to allow multiplexing in both steps. Multiplexes were performed using the Type-IT Microsatellite PCR kit (Qiagen). The first multiplex step used all primer pairs (unlabelled) and had a final reaction volume of 20 uL, including 5 uL of template DNA. The second multiplex step involved six separate combinations of labelled primer pairs. Final reaction volume for the 2^nd^ step mplexes were 10 uL, including 2.5 uL of 1:100 diluted PCR product from the first step.

Final primer concentrations for the first round of amplification were as follows: UTX1, SRY1, Ceb01, Ceb07, Ceb09, Ceb11, Ceb130 at 0.1 uM each; d7s794, d3s1766, d12s372, Ceb03, Ceb10, Ceb120, Ceb127, Ceb128 at 0.15 uM each; Ceb02, Ceb04, Ceb08, Ceb12 at 2.5 uM each; Ceb105, Ceb115, Ceb119 at 3.5 uM each.

Final concentrations for the 6 combinations of primers for the second round of amplification were as follows:

C1 – d7s794 (0.15 uM), d3s1766 (0.15 uM), d12s372 (0.15 uM);

C2 – Ceb09 (0.15 uM), Ceb10 (0.15 uM), Ceb119 (0.3 uM), Ceb120 (0.15 uM);

C3 – Ceb01 (0.1 uM), Ceb03 (0.15 uM), Ceb08 (0.15 uM), Ceb115 (0.3 uM);

C4 – Ceb02 (0.15 uM), Ceb04 (0.15 uM), Ceb07 (0.1 uM), UTX1 (0.15 uM), UTY2 (0.15 uM), SRY1 (0.15 uM);

C5 – Ceb105 (0.3 uM), Ceb127 (0.15 uM), Ceb128 (0.15 uM);

C6 – Ceb11 (0.1 uM), Ceb121 (0.15 uM), Ceb130 (0.15 uM).

PCR products were generated using S1000 Thermal Cyclers (BIO-RAD). The first round of amplification was done using a multiplex-touchdown PCR protocol. There was an initial denaturing step at 95°C for 5 minutes, followed by 36 cycles of denaturing at 95° for 30 seconds, annealing (65°-57°) for 90 seconds, and extension at 72° for 30 seconds. Final extension was at 72° for 30 minutes. The annealing temperature in the first two cycles was 65°, followed by 64° (2 cycles), 63° (2 cycles), 62° (2 cycles), 61° (2 cycles), 60° (2 cycles), 59° (2 cycles), 58° (2 cycles), and 57° (20 cycles) for a total of 36 cycles. Thermocycler programs for the second round of amplification are available in **Table 1**.

PCR products were analyzed on an ABI PRISM 3100 Genetic Analyser using an internal size standard (ROX labelled HD400) and GENEMAPPER software (Applied Biosystems).

Each DNA sample was analyzed at a minimum in triplicate, with additional amplification if the sample was of low quality (e.g., low concentration of DNA). Heterozygotes were scored if each allele was seen at least twice, while homozygotes were scored after seeing an allele at least three times in the absence of a second allele. If there was any uncertainty as to the scoring, additional samples were run to clarify the genotype.

Under the new 2-step PCR protocol, seven primer pairs either did not reliably produce PCR products (Ceb02, Ceb115, Ceb130, D7s794, D3s1766, D12s371) or failed to produce any measurable product (UTY2). Genotypes were thus limited to 14 autosomal and two sex-specific microsatellite markers.

Parentage assignment

Parentage analysis was carried out using a likelihood-based approach with CERVUS 3.0.7 software (Kalinowski et al. 2007). Parentage analysis simulations were carried out with the following settings: 10 000 iterations, 1% loci mistyped, 95% of candidate parents sampled, eight candidate fathers, and a minimum of 7 loci typed. Parentage assignments were determined using strict confidence levels of 95%.

Males of at least 6 years of age were included as candidate fathers if they resided in the same group as an offspring’s mother around the time of the offspring’s conception. We excluded 2 offspring with unsampled mothers, and the remaining 104 offspring were genotyped at an average of 13.25 loci (range: 9-14). The average proportion of sampled parents was 0.9893 and the average number of candidate fathers was 8.

For all 104 newly genotyped capuchins with sampled mothers, CERVUS assigned a father from within the pool of candidate males. Most of these assigned parent-offspring trios had zero mismatches (97.1%), with three trios having only 1 mismatch. In one case, two candidate fathers had zero mismatches with an offspring. This case involved related candidate fathers, with the second male being the paternal half-brother of the assigned father.

*Table 1: Thermocycler programs for second round of multiplexes. A touchdown PCR approach was used, decreasing the annealing temperature by 1°C after two cycles.*

| Temperature (°C) | Time (MM:SS) | Cycles | Combo-specific annealing temperatures |  |
| --- | --- | --- | --- | --- |
| 95 | 5:00 | x 1 |  |  |
| 95 | 0:30 |  |  |  |
| * | 1:30 | x 2 | C1 (55), C2 (63), C3 (61), C4 (58), C5 (63), C6 (62) |  |
| 72 | 0:30 |  |  |  |
| 95 | 0:30 |  |  |  |
| * | 1:30 | x 2 | C1 (54), C2 (62), C3 (60), C4 (57), C5 (62), C6 (61) |  |
| 72 | 0:30 |  |  |  |
| 95 | 0:30 |  |  |  |
| * | 1:30 | x 2 | C1 (53), C2 (61), C3 (59), C4 (56), C5 (61), C6 (60) |  |
| 72 | 0:30 |  |  |  |
| 95 | 0:30 |  |  |  |
| * | 1:30 | x 31 | C1 (52), C2 (60), C3 (58), C4 (55), C5 (60), C6 (59) |  |
| 72 | 0:30 |  |  |  |
| 72 | 30:00 | x 1 |  |  |

**Preliminary analysis of seasonality in sociality**

In our first preliminary models, we modelled the effect of seasonality on sociality by including month of data collection (Month, n=12) as a fixed effect. We included the random effect of the identity of subject (ID, n=376), and ran our model using a weakly informative prior for the random effects (V=1, nu=0.02).

Such models, however, assume that months are independent from each other, when months closer to each other are likely to be similar. Visual inspection of the effect of month indeed showed cyclical seasonality (**Supplementary Figure 1a and 2a**).

Based on this, in our second set of preliminary models, we accounted for seasonality using a simple sine wave of one cycle. To accomplish this, we transformed the month variable as follows:

$Month^{'}=\frac{2\pi*Month}{12}$ ,

where *month* took an integer value from 1 to 12. We then included both the sine and the cosine of this transformed variable as fixed effects. We additionally included month as a random effect in order to model deviations from the sine wave. However, V_Month_ only captured a small proportion of the variance on the latent scale for time spent social (posterior mode: 0.0108, 95 HPDI: 0.0051, 0.0401) and number of partners (posterior mode: 0.0183, 95HPDI: 0.0073, 0.0564) models, suggesting that our sine wave captured most of the monthly variation.

We found evidence that both the sine (social vs alone 95 CI: 0.0903 0.2240; number of partners 95 CI: 0.0582, 0.1766) and the cosine (social versus alone 95 CI: -0.2268, -0.1010; number of partners 95 CI: -0.2197, -0.1040) of our transformed term had an effect on our sociality measure (**Supplementary Figure 1b and 2b**), and so they are included as fixed effects in all models reported in the main text.

**SI Figure 1: Fixed effects estimates for preliminary models for seasonality (social versus alone).** Output is on the logit scale. All models were run in quadruplicate in order to check for convergence. The output presented is from the first chain for each model.

**SI Figure 2: Fixed effects estimates for preliminary models for seasonality (number of partners).** Output is on the log scale. All models were run in quadruplicate in order to check for convergence. The output presented is from the first chain for each model.

**SI Figure 3: Proportion of variance explained by variance components, categorized by prior specification for random effects.** 95% highest posterior density intervals are plotted. Dots represent the posterior mode. Output is on the latent scale without V_FE_ accounted for in V_P_. All models were run in quadruplicate in order to check for convergence. The output presented is from the first chain for each model.

Estimates generated from the *QGglmm* package for model output run with *MCMCglmm* using family = “multinomial2” depend on the n.obs parameter. This specifies the number of trials for a binomial model. This is because the model estimates the number of successes for a particular number of trials. As the number of trials go up, the standard error around those estimates will go down. We use the average number of scans per individual per month (n.obs = 32) when reporting estimates in the main text. However, we show here the consequences of changes to the n.obs parameter (**SI Figure 4**). We plot across the range of sampling values from our dataset, which had the upper limit of 317 scans for one individual in a particular month.

**SI Figure 4: Repeatability and heritability estimates, categorized by number of trials for binomial model with logit link function**. Output is on the observed data scale without V_FE_ accounted for in V_P_. Repeatability and heritability were calculated using the QGicc() and QGparams() functions respectively from the QGglmm package (de Villemereuil 2016; de Villemereuil et al. 2016). Repeatability estimates are based on $\frac{(V_{ID}+V_{M}+V_{A})}{V_{P}}$.

***SI Figure 5****:* ***Proportion of variance explained by variance components, categorized by fixed effects structure.*** *Models were running using an inverse Wishart prior for the variance components (V=1, nu=0.002). 95% highest posterior density intervals are plotted. Dots represent the posterior mode. Output is on the latent scale. All models were run in quadruplicate in order to check for convergence. The output presented is from the first chain for each model and without V_FE_ included in V_P_. All models in b) also include the natural log of the number of scans (sampling effort).*

******

***SI Figure 6****:* ***Proportion of variance explained by variance components, categorized by fixed effects structure.*** *Models were running using an inverse Wishart prior for the variance components (V=1, nu=0.02). 95% highest posterior density intervals are plotted. Dots represent the posterior mode. Output is on the latent scale. All models were run in quadruplicate in order to check for convergence. The output presented is from the first chain for each model and without V_FE_ included in V_P_. All models in b) also include the natural log of the number of scans (sampling effort).*

References

Arandjelovic M, Guschanski K, Schubert G, Harris TR, Thalmann O, Siedel H et al. (2009) Two-step multiplex polymerase chain reaction improves the speed and accuracy of genotyping using DNA from noninvasive and museum samples. *Mol Ecol Resour*.

Cadamuro VC, Bouakaze C, Croze M, Schiavinato S, Tonasso L, Gérard P et al. (2015) Determined about sex: Sex-testing in 45 primate species using a 2Y/1X sex-typing assay. *Forensic Sci Int Genet* **14**: 96–107.

de Villemereuil P (2016) *How to use the QGglmm package ?*

de Villemereuil P, Schielzeth H, Nakagawa S, Morrissey M (2016) General methods for evolutionary quantitative genetic inference from generalized mixed models. *Genetics* **204**: 1281–1294.

Di Fiore A (2005) A rapid genetic method for sex assignment in non-human primates. *Conserv Genet* **6**: 1053–1058.

Godoy I, Vigilant L, Perry SE (2016) Cues to kinship and close relatedness during infancy in white-faced capuchin monkeys, *Cebus capucinus*. *Anim Behav* **116**: 139–151.

Kalinowski ST, Taper ML, Marshall TC (2007) Revising how the computer program CERVUS accommodates genotyping error increases success in paternity assignment. *Mol Ecol*.

Morin PA, Chambers KE, Boesch C, Vigilant L (2001) Quantitative polymerase chain reaction analysis of DNA from noninvasive samples for accurate microsatellite genotyping of wild chimpanzees (Pan troglodytes verus). *Mol Ecol* **10**: 1835–1844.

Muniz L, Perry S, Manson JH, Gilkenson H, Gros-Louis J, Vigilant L (2006) Father-daughter inbreeding avoidance in a wild primate population. *Curr Biol* **16**: R156–R157.

Muniz L, Vigilant L (2008) Isolation and characterization of microsatellite markers in the white-faced capuchin monkey (*Cebus capucinus*) and cross-species amplification in other New World monkeys. *Mol Ecol Resour* **8**: 402–405.

Nsubuga AM, Robbins MM, Roeder AD, Morin PA, Boesch C, Vigilant L (2004) Factors affecting the amount of genomic DNA extracted from ape faeces and the identification of an improved sample storage method. *Mol Ecol*.

Roeder AD, Archer FI, Poinar HN, Morin PA (2004) A novel method for collection and preservation of faeces for genetic studies. *Mol Ecol Notes*.

Villesen P, Fredsted T (2006) Fast and non-invasive PCR sexing of primates: Apes, Old World monkeys, New World monkeys and Strepsirrhines. *BMC Ecol* **6**.
